# N^6^-Methyladenosine RNA Modification: A Potential Regulator of Stem Cell Proliferation and Differentiation

**DOI:** 10.3389/fcell.2022.835205

**Published:** 2022-04-04

**Authors:** Bo Wei, Meiyu Zeng, Jing Yang, Shuainan Li, Jiantao Zhang, Nan Ding, Zhisheng Jiang

**Affiliations:** ^1^ Research Lab of Translational Medicine, Hengyang Medical School, University of South China, Hengyang, China; ^2^ Key Laboratory for Arteriosclerology of Hunan Province, Human International Scientific and Technological Cooperation Base of Arteriosclerotic Disease, Institute of Cardiovascular Disease, Hengyang Medical College, University of South China, Hengyang, China; ^3^ Institution of Pathogenic Biology, Hengyang Medical School, University of South China, Hengyang, China

**Keywords:** N^6^-methyladenosine, RNA modification, stem cell, proliferation, differentiation

## Abstract

Stem cell transplantation (SCT) holds great promise for overcoming diseases by regenerating damaged cells, tissues and organs. The potential for self-renewal and differentiation is the key to SCT. RNA methylation, a dynamic and reversible epigenetic modification, is able to regulate the ability of stem cells to differentiate and regenerate. *N* ^6^-methyladenosine (m^6^A) is the richest form of RNA methylation in eukaryotes and is regulated by three classes of proteins: methyltransferase complexes, demethylase complexes and m^6^A binding proteins. Through the coordination of these proteins, RNA methylation precisely modulates the expression of important target genes by affecting mRNA stability, translation, selective splicing, processing and microRNA maturation. In this review, we summarize the most recent findings on the regulation of m^6^A modification in embryonic stem cells, induced pluripotent stem cells and adult stem cells, hoping to provide new insights into improving SCT technology.

## Introduction

Stem cells are unique cell populations possessing proliferation and differential ability, which have been identified in embryo, reprogrammed somatic cells and many adult tissues, including muscle, brain, bone marrow, blood and fat, and play a vital role in the field of regenerative medicine (Gurusamy et al., 2018; Belenguer et al., 2021; Ding et al., 2021). *In vitro*, stem cells can differentiate into specific cell lineages in response to special inducers, allowing for the replacement of lost cells, providing tropic support and modulating inflammation (Gnecchi et al., 2012; Mouhieddine et al., 2014; Ji et al., 2018; Cheng et al., 2019). With advances in stem cell transplantation (SCT) technology, the process of treating diseases associated with poor regenerative abilities and limited amounts of endogenous adult stem cells (ASCs), such as cardiovascular, nervous and sports system diseases, has progressed (Beitnes et al., 2011; Ma et al., 2013; Trounson and Mcdonald, 2015; Gallo et al., 2016; Gorecka et al., 2018). Increase of the ability to self-renewal and differentiate into specific cell lineages of stem cells will be of great significance for improving the SCT efficiency.

**FIGURE 1:**
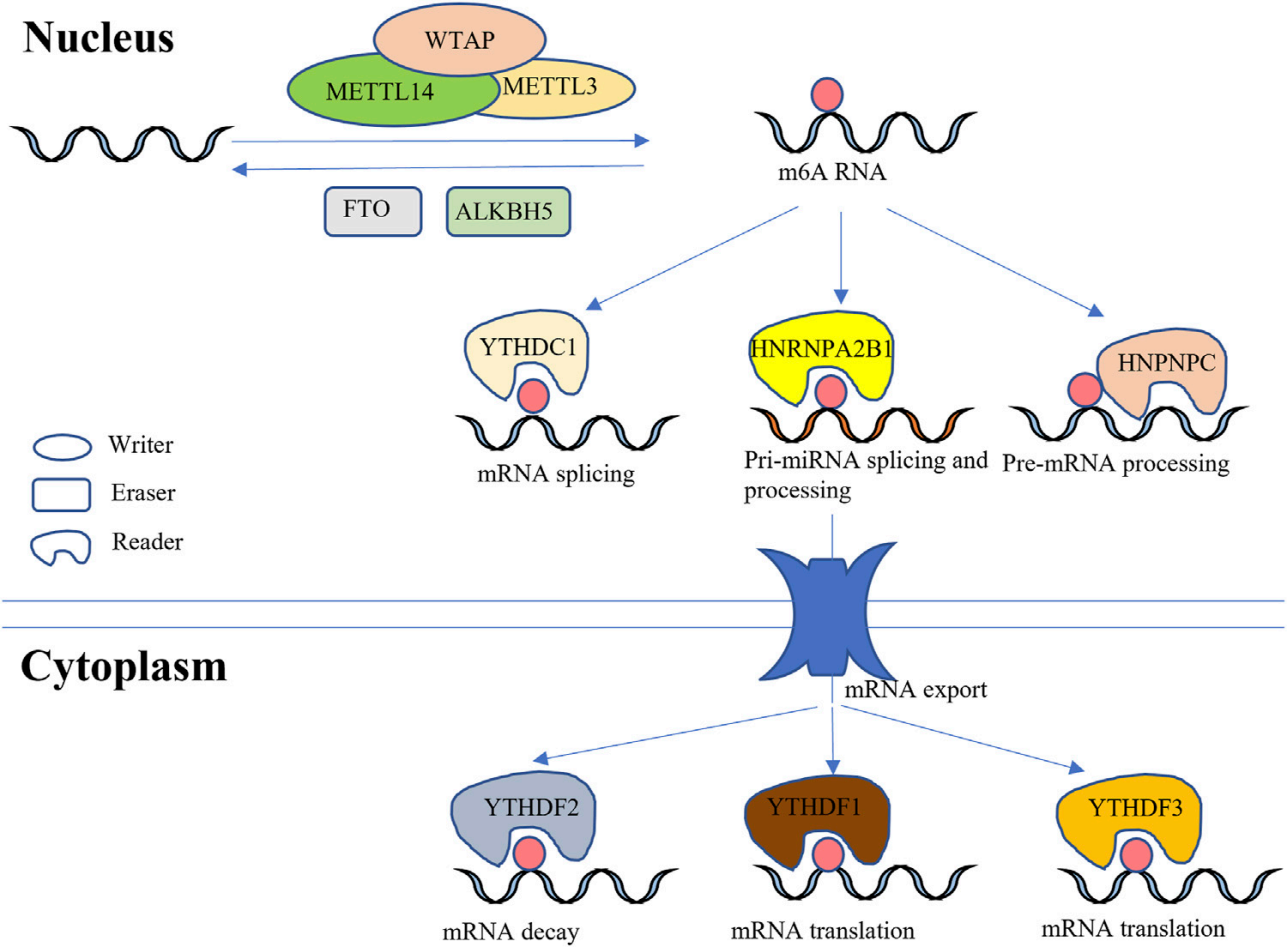
Coordination between writer, eraser and reader. The ellipse represents the writer, which possesses the ability to catalyze RNA into m^6^A-containing RNA. The rectangle indicates the eraser, which moves m^6^A, thereby downregulating m^6^A-RNA. Precise coordination between the writer and eraser allows for dynamic and reversible regulation of m^6^A levels in eukaryocytes. The irregular shape represents the reader, which binds m^6^A and modulates expression of important genes. Both the nucleus and cytoplasm contain reader, and different readers play unique roles in the physiological functioning of organisms. Some readers, notably YTHDF1 and YTHDF3, promote the expression of specific genes, whereas YTHDF2 has the opposite effect, decreasing mRNA levels.

RNA methylation, which was first identified in Novikoff hepatoma cells in 1970, influences various cell processes (C. Desrosiers et al., 1974). In 2011, RNA methylation once again received the attention of scientists as a reversible and dynamic epigenetic modification (Jia et al., 2011). Although more than 100 types of chemical modifications, such as N^1^-methyladenosine (m^1^A), N^6^, 2′-O-dimethyladenosine (m^6^A_m_), as well as *N* ^6^-methyladenosine (m^6^A), have been identified in cellular RNAs, m^6^A has been considered as the most abundant modification in eukaryotes (Roundtree et al., 2017), which is common in 3′ untranslated regions (UTRs), internal long exons and stop codons. It has been reported that the function of m^6^A is largely dependent on modifications around 3′ UTRs (Meyer et al., 2012; Ke et al., 2015; Lin et al., 2016). The proteins that regulate m^6^A modification levels have been named methyltransferase complexes (writer), demethylase complexes (eraser) and binding protein complexes (reader) (Figure 1). To date, three main methyltransferases, METTL3, METTL14 and WTAP, as well as a newly reported METTL5, have been found to catalyze RNA m^6^A (Ignatova et al., 2020). In addition, two kinds of important demethylases, FTO and ALKBH5, possess the ability to remove RNA m^6^A (Wu et al., 2019). Coordination of the writer and eraser allows for dynamic changes in m^6^A levels according to developmental stage and physiological conditions. However, the reader can only modulate cell processes when it binds m^6^A sites. To date, the YTH domain family, HNRNPC family and HNRNPA2B1 have been identified as binding proteins that play different roles in m^6^A functioning (Ji et al., 2018). Among YTH domain family members, YTHDF2 was the first found to bind with m^6^A-RNA to regulate mRNA degeneration (Mapperley et al., 2021). YTHDF1 and YTHDF3 can improve the translation efficiency of m^6^A-mRNA upon binding (Wang et al., 2015; Tiwary, 2020). In addition, YTHDC1 (initially referred as YT521-B), a binding protein in the cell nucleus, can regulate RNA splicing (Xu et al., 2014; Roundtree and He, 2016). HNRNPC family members include HNRNPC and HNRNPG, which can switch the secondary structure of mRNA (Liu et al., 2017). Unlike the YTH domain family and HNRNPC family, HNRNPA2B1 can recognize m^6^A sites and further regulate the splicing and processing of pri-miRNA (Alarcon et al., 2015).

**FIGURE 2:**
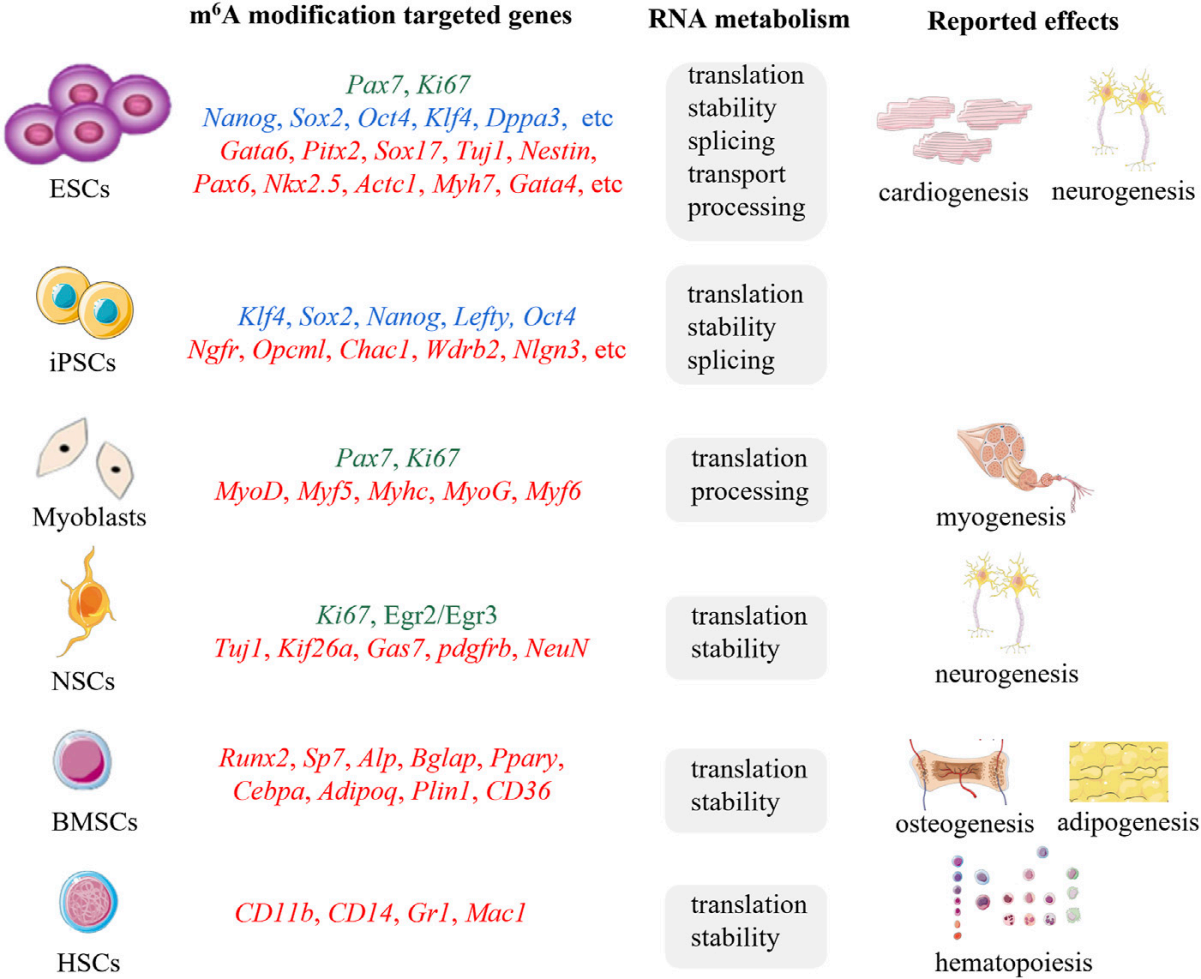
The m^6^A modifications targeted genes, associated RNA metabolic mechanisms, and reported effects of ESCs, iPSCs, myoblasts, NSCs, BMSCs, HSCs. Proliferation associated genes (green), pluripotency related genes (blue), differentiation associated genes (red).

M^6^A methylation have shown powerful bioregulatory functions in RNA metabolism including pre-mRNA splicing, mRNA translation, stability, and transport, 3′-end processing, and non-coding RNA processing (Sun et al., 2019; Oerum et al., 2021), which is involved in the regulation of a series of cellular processes such as cell fate decision, cell cycle regulation, cell proliferation and differentiation, indicating its pivotal roles in embryonic development, hematopoietic development, neurogenesis, stress responses, sex determination, tumorigenesis and so on (Liu et al., 2018; Jiang et al., 2021). Increasing evidences have displayed that m^6^A methylation possesses the ability to regulate proliferation and differentiation of stem cells by modulating the expression of pluripotency, proliferation or lineage-specific genes (Figure 2) (Lin and Gregory, 2014; Li J. et al., 2021), implying great application prospects for SCT. In this review, we provide a comprehensive summary of the biological functions of m^6^A in stem cell proliferation and differentiation to facilitate the improvement of SCT technology.

## THE Role of m^6^A in Embryonic Stem Cells

Embryonic stem cells (ESCs) are derived from the inner cell mass of the developing blastocyst possessing the ability to self-renewal and form any fully differentiated cell of the body (Biswas and Hutchins, 2007; Young, 2011). The cell fate of ESCs between proliferation and differentiation is regulated by multiple pathways that orchestrate gene expression (Table 1). The self-renewal capacity of ESCs is maintained by activating the pluripotency genes such as *Oct4*, *Nanog*, *Sox2*, *Dppa3*, and *Klf4*, as well as suppressing lineage-specific genes (Yeo and Ng, 2013). *Ki67* is the most important marker associated with stem cells proliferation. Additionally, a few developmental regulators associated genes, including *Gata6*, *Pitx2*, *Sox17*, *Cdx2* were involved in the ESCs differentiation except some lineage-specific ones (Wang et al., 2014). Many ESCs transcripts of developmental regulators and lineage priming regulators contain m^6^A, suggesting that it plays a vital role in the decision of ESC fate.

**TABLE 1:** RNA methylation regulators and their functions in different types of stem cells.

Stem Cell types	Regulatory proteins		Effects to proliferation and differentiation	References
mESCs	Writer	METTL3	*Mettl3* deficiency inhibited proliferation and promoted differentiation related with HuR and microRNA pathways	Wang et al. (2014)
			*Mettl3* depletion enhanced proliferation and suppressed differentiation	Batista et al. (2014)
			*Mettl3* ablation blocked differentiation and promoted proliferation in mESCs, but played an opposite role in EpiSCs	(Geula et al. (2015); Zhao and He (2015))
			Catalyzing m^6^A of lincRNA 1281 to promote differentiation without influencing proliferation	Yang et al. (2018)
		METTL5	Catalyzing m^6^A in 18S rRNA to trigger proliferation and differentiation	Ignatova et al. (2020)
		METTL14	*Mettl14* deficiency inhibited proliferation and promotes differentiation	Wang et al. (2014)
hESCs	Eraser	ALKBH5	Demethylating m^6^A of *Sox2* to promote differentiation without affecting proliferation	Chen C et al. (2021)
			Decreasing global m^6^A level to inhibit cardiomyocyte differentiation	Han et al. (2021)
mESCs	Reader	YTHDF3	*Ythdf3* loss displayed increased cell proliferation and accelerated cardiac differentiation	Wang Y et al. (2021)
		YTHDF1	*Ythdf1* depletion inhibited proliferation and cardiac differentiation	
		YTHDC1	Recognizing m^6^A on LINE1 RNAs to promote proliferation and differentiation	Chen X et al. (2021)
		HNRNPA2/B1	Promoting proliferation and differentiation in a METTL3-dependent manner	Kwon et al. (2019)
	other	PRMT1	Arginine methylation of METTL14 to promote proliferation and endoderm differentiation	Liu T et al. (2021); Wang P et al., (2021)
		Zc3h13	Facilitating nuclear m^6^A methylation to promote proliferation and inhibit differentiation	Wen et al. (2018)
		Zfp217	Interacting with METTL3 to suppress m^6^A methylation to inhibit differentiation in undifferentiated state cells and trigger differentiation in differential cells	Lee et al. (2016); Aguilo and Walsh. (2017)
piPSCs	Writer	METTL3	*Mettl3* silence impaired proliferation and triggered differentiation by targeting SOCS3/JAK2/STAT3 pathway	Wu et al. (2019)
iPSCs	Reader	YTHDF2/3	*Ythdf2/3* deficiency inhibited proliferation	Liu et al. (2020)
		YTHDF2	Destabilizing m^6^A-modified transcripts to restrain differentiation	Heck et al. (2020)
	Other	SMAD2/3	Interacting with METTL3–METTL14–WTAP complex to trigger differentiation	Bertero et al. (2018)
		MATR3	Enhancing proliferation and differentiation by binding to the *Oct4* and *Ythdf1* promoters	Pollini et al. (2021)
Myoblasts	Writer	METTL3	Inhibiting proliferation and promoting myogenic differentiation through Notch signaling pathway	Chen J. N et al. (2019); Liang et al. (2021)
			Inhibiting myogenic differentiation *via* repressing muscle specific miRNAs	Diao et al. (2021)
		METTL14	Inhibiting proliferation and promoting differentiation	Zhang et al. (2020)
	Eraser	FTO	*Fto* silence impaired myogenic differentiation *via* mTOR-PGC-1α pathway-mediated mitochondria biogenesis	Wang et al. (2017)
			*Fto* depletion inhibited proliferation by controlling CCND1 expression depending on YTHDF2	Deng et al. (2021)
			Promoting proliferation and differentiation through the focal adhesion pathway	Huang et al. (2020)
	Reader	IGF2BP1	Inhibiting proliferation and promoting differentiation *via* target genes associated with muscle development	Zhang et al. (2020)
NSCs	Writer	METTL3	*Mettl3* depletion reduced proliferation and neurogenesis *via* modulating histone methyltransferase Ezh2	(Chen J et al. (2019))
		METTL14	Essential for proliferation and maintenance of NSCs in an undifferentiated state through histone modifications	(Wang H et al. (2018))
	Eraser	FTO	*Fto* knockdown inhibited proliferation and differentiation *via* brain derived neurotrophic factor pathway	Li et al. (2017)
			*Fto* ablation transiently enhanced proliferation and neuronal differentiation, but inhibited neurogenesis in a long time *via* Pdgfra/Socs5-Stat3 pathway	Cao et al. (2020)
	Reader	FMRP	Binding m^6^A-tagged mRNAs to facilitate nuclear export through CRM1 and promote neural differentiation	Edens et al. (2019)
BMSCs	Writer	METTL3	*Mettl3* loss led to impaired bone formation, incompetent osteogenic differentiation *via* PTH/Pth1r, PI3K-Akt pathway	(Wu et al. (2018); Tian et al. (2019))
			Promoting angiogenesis to improve bone regeneration	Tian et al. (2019)
			Triggering osteoblast differentiation and bone formation *via* m^6^A modification of precursor-miR-320/RUNX2	Yan et al. (2020)
			Facilitating osteogenic differentiation *via* the LINC00657/miR-144-3p/BMPR1B axis	Peng et al. (2022)
			Inhibiting osteogenesis by enhancing m^6^A methylation of MYD88 and subsequently activating NF-κB	Yu et al. (2020)
			Suppressing adipogenic differentiation *via* the JAK1/STAT5/C/EBPβ pathway depending on YTHDF2	Yao et al. (2019)
			Catalyzing m^6^A methylation of AKT1 to reduce adipogenesis	Pan et al. (2021)
		METTL14	Triggering m^6^A methylation of PTPN6 and activating Wnt signaling pathway to enhance cell proliferation and osteogenic differentiation	Cheng et al. (2021)
	Eraser	FTO	Facilitating adipogenesis rather than osteogenesis *via* demethylating mRNA of PPARγ	Shen et al. (2018)
			Triggering osteogenic differentiation through demethylating *Runx2* mRNA without influencing proliferation	Wang Z et al. (2021)
		ALKBH5	Inhibiting osteogenic capacity by improving mRNA decay rate of PRMT6 *via* PI3K/AKT pathway	Li Z et al. (2021)
			Coordinating with METTL3 to regulate osteogenic differentiation *via* NF-κB signaling	Yu et al. (2020)
			Promoting osteoblast differentiation through modulating *Runx2* mRNA stability	Feng et al. (2021)
	Reader	YTHDF1	Targeting ZNF839 to promote osteogenic differentiation	Liu X et al. (2021)
		YTHDF2	Facilitating the degradation of JAK1 to inhibit JAK/STAT signaling pathway, thus suppressing the differentiation	Yao et al. (2019)
HSCs	Writer	METTL3	Inhibiting proliferation and myeloid differentiation by targeting m^6^A in MYC and affecting AKT pathway	(Vu et al. (2017); Lee et al. (2019))
		METTL14	Blocking differentiation into myeloid cells through SPI1-METTL14-MYB/MYC signaling axis	Weng et al. (2018)
	Reader	YTHDF2	Inhibiting cells expansion	(Huang and Broxmeyer (2018); Li et al. (2018))

### Writers

Early reports about the role of METTL3 and METTL14 in the self-renewal and differentiation of ESCs was somewhat controversial. Wang *et al.*, reported that either *Mettl3* or *Mettl14* deficiency in mouse ESCs (mESCs) impaired self-renewal, and enhanced the expressional levels of developmental genes such as *Fgf5* and *Cdx2*, suggesting the loss of m^6^A levels inhibited self-renewal, and promoted differentiation of mESCs (Wang et al., 2014). On the contrary, another study revealed that genetic inactivation or depletion of *Mettl3* in mESCs and human ESCs (hESCs) prolonged *Nanog* expression upon differentiation to make the ESCs incapable of differentiating into specific lineages (Batista et al., 2014). However, the differences of m^6^A modification on proliferation and differentiation in ESCs were explained by Geula et al., and others that the m^6^A modification could decrease mRNA stability of naïve pluripotency-promoting genes or pro-differentiation genes depending on the pluripotent state of the ESCs, i.e., naive or primed (Geula et al., 2015; Zhao and He, 2015). In naïve pluripotent mESCs, *Mettl3* knockout drive the transition from the naïve state into the primed epiblast stem cells (EpiSCs), which indicated a more differentiated state. Naïve mESCs presented increased pluripotency upon *Mettl3* knockdown, whereas primed EpiSCs displayed enhanced propensity to lineage differentiation upon m^6^A depletion. Therefore, *Mettl3* depletion blocked differentiation and promoted proliferation in mESCs, but played an opposite role in EpiSCs (Geula et al., 2015; Zhao and He, 2015). METTL3-mediated m^6^A modification of lincRNA 1281 is critically required for proper differentiation of mESCs by sequestering its interaction with pluripotency-related let-7 family microRNAs without influencing its proliferation (Yang et al., 2018). METTL5 also occupied an important role in m^6^A modification, which catalyzed m^6^A in 18S rRNA at position A_1832_ to enhance global translation rate, promoted the self-renewal and differentiation of mESCs (Ignatova et al., 2020).

### Erasers

Chen et al. developed a targeted RNA m^6^A erasure system to demethylate RNAs site-specifically in hESCs, thus modulating individual m^6^A modification in ESCs with precise temporal control (Chen X. et al., 2021). The fusion of the catalytic domain of ALKBH5 with a stably transfected, doxycycline-inducible dCas13a precisely and reversibly demethylated the targeted m^6^A site of *Sox2*, which significantly increased the amount and stability of corresponding transcripts and facilitated the differentiation of hESCs without affecting the self-renewal capacity (Chen X. et al., 2021). In addition, overexpression of ALKBH5 remarkably blocked cardiomyocyte differentiation of hESCs by down-regulating the expression of KDM5B and RBBP5, two downstream targets for ALKBH5 in cardiac-committed hESCs (Han et al., 2021).

### Readers

YTHDF1, YTHDF3, YTHDC1 and HNRNPA2/B1 are readers reported to be involved in the regulation of m^6^A in ESCs. Interestingly, YTHDF1 and YTHDF3 not only displayed differential roles in regulating the pluripotency of ESCs, but also showed converse contribution to the cardiac differentiation (Wang S. et al., 2021). Depletion of *Ythdf3* in mESCs led to loss of pluripotency with accelerated cardiac differentiation through facilitating the cardiomyocyte-specific genes expression and displayed increased cell proliferation. While, *Ythdf1* loss resulted in marked impairment of cardiomyocytes differentiation, as well as slightly decreased proliferation. Remarkably, YTHDF3 appears to mediate cellular differentiation partially by suppressing YTHDF1, demonstrating the contrasting but interrelated roles of YTHDF1 and YTHDF3 in determination of cell fate (Wang S. et al., 2021). Additionally, YTHDC1 could recognize the m^6^A-marked LINE1 RNAs in the nucleus and regulate the formation of the LINE1-NCL-KAP1 complex to modulate the RNA scaffold, thus ensuring the appropriate transcriptome and developmental potency of mESCs and early embryos (Chen C. et al., 2021). HNRNPA2/B1 was essential for early embryonic development by promoting the pluripotency-related gene expression depending on the METTL3-mediated m^6^A RNA methylation (Choi et al., 2013; Kwon et al., 2019).

### Other Regulatory Proteins

The m^6^A modification level in ESCs were regulated by multiple proteins. Protein arginine methyltransferase 1 (PRMT1) can increase the global m^6^A RNA level (Liu X. et al., 2021; Wang Z. et al., 2021). PRMT1 was able to interact with and methylate METTL14 in its disordered C terminal region at R255 to enhance its RNA methylation activity and promoted the binding of the METTL3/METTL14 complex to substrate RNA, thereby triggering global m^6^A modification and mESCs endoderm differentiation (Liu X. et al., 2021; Wang Z. et al., 2021). Zinc-finger protein Zc3h13 played a negative role in the m^6^A methylation, which directly interacted with WTAP to form a Zc3h13-WTAP-Virilizer-Hakai complex to regulate RNA m^6^A methylation in the nucleus. *Zc3h13* knockdown markedly impaired self-renewal and promoted mESCs differentiation (Wen et al., 2018). Interestingly, Zinc finger protein 217 (Zfp217) displayed significant discrepancy in affecting the level of m^6^A methylation owning to the cell state differences, which could modulate m^6^A deposition on their transcripts *via* sequestering the METTL3, thus hindering METTL3 binding to RNAs (Lee et al., 2016; Aguilo and Walsh, 2017). mESCs in the undifferentiated state showed high level of ZFP217, which significantly suppressed METTL3 methyltransferase activity, preventing core ESC transcripts from aberrant methylation. However, in differential mESCs, ZFP217 contents and its target genes promptly decreased, allowing the METTL3 to release and catalyze m^6^A methylation at the remaining pluripotency transcripts, thus triggering ESC differentiation (Aguilo et al., 2015; Lee et al., 2016).

## The Role of m^6^A in Induced Pluripotent Stem Cells

Induced pluripotent stem cells (iPSCs) were originally generated in 2006 by successfully transferring four transcription factors *Oct4*, *Sox2*, *Klf4*, and *c-Myc* into adult somatic cells to reprogram them (Takahashi et al., 2007; Park et al., 2008). Compared to ESCs, iPSCs derived from adult somatic tissues, such as skin, blood, and urine, displayed no immune rejection when transplanted autologously, which made iPSCs an extraordinary candidate for personalized medicine. M^6^A modification remarkably influences the pluripotency and differentiation of iPSCs (Table 1).

### Writers

Owning to the great similarity in genome and physiological characteristics, porcine iPSCs (piPSCs) have become an important study model. *Mettl3* deficiency markedly inhibited self-renewal and promoted differentiation of piPSCs. Mechanistically, METTL3 silence caused lowered m^6^A levels of JAK2 and SOCS3, which induced the alteration of JAK2 and SOCS3 protein expression, thus inhibiting JAK2–STAT3 pathway to affect proliferation and differentiation of piPSCs (Wu et al., 2019).

### Readers

YTHDF2 and YTHDF3 are two readers reported to be required for the reprogramming of somatic cells into iPSCs. Both the YTHDF2-CCR4-NOT and YTHDF3-PAN2-PAN3 deadenylase complex could promote the mRNA clearance of somatic genes, such as Tead2 and Tgfb1, thus conducting the reprograming (Liu et al., 2020). The cell proliferation of iPSCs was significantly decreased upon Ythdf2/3 deficiency (Liu et al., 2020). In addition, YTHDF2 could destabilize m^6^A-modified transcripts related with neural development, thus restraining differentiation in iPSCs (Heck et al., 2020).

### Other Regulatory Proteins

SMAD2/3 and Matrin3 (MATR3) are two proteins associated with the m^6^A methylation in iPSCs. SMAD2/3 interacted with METTL3–METTL14–WTAP complex to mediate the conversion of adenosine to m^6^A on RNA, which facilitated the destabilization of SMAD2/3 targeted genes such as pluripotency factor gene *Nanog*, thus leading to timely exit from pluripotency and neuroectodermal differentiation (Bertero et al., 2018). MATR3 acted as a nuclear RNA/DNA-binding protein to regulate gene expression by stabilizing target RNAs. MATR3 maintained pluripotency in human iPSCs mainly in two ways. For one thing, MATR3 binded to the *Oct4* and *Ythdf1* promoters to facilitate their expression. For another, MATR3 was recruited on ribosomes to regulate the translation of various specific transcripts such as *Nanog* and *Lin28a* through direct binding to keep their stabilization. MATR3 deficiency hampered both the proliferation and differentiation of human iPSCs (Pollini et al., 2021).

## The Role of m^6^A in ASCs

### The Role of m^6^A in Myoblasts/Muscle Stem Cells

Skeletal muscle degeneration is associated with various conditions, including developmental disorders, muscular dystrophies, neuromuscular degenerative diseases, cardiac diseases, and aging (Emery, 2002; Negroni et al., 2015; Chal and Pourquie, 2017). Skeletal myoblasts derive from satellite cells (progenitor cells) which are responsible for skeletal muscle growth and repair. Following muscle damages, the satellite cells exit cell cycle to differentiate into myoblasts, which further proliferate and fuse to myotubes and fresh muscle fibers to repair tissues. The phase of myogenic differentiation mainly includes early stage and late stage. *MyoD* and *Myf5* have been considered as early-stage markers, whereas *MyoG*, *Mrf4* (*Myf6*), and *MyHC* have been regarded as later-stage markers (Chen et al., 2019). Myoblasts act as the first candidates for stem cell therapy in muscle repair owing to their easy to obtain from muscle biopsies and autologous transplantation characteristics (Negroni et al., 2015; Rikhtegar et al., 2019). Therefore, enhancing the myogenic capacity of myoblasts holds great promise for alleviating skeletal muscle degenerative diseases.

#### Writers

The discovery that m^6^A RNA methylation was directly involved in the regulation of myogenic differentiation was performed in murine C2C12 myoblasts. METTL3 possessed a vital role in controlling the transition of muscle stem cells/myoblasts to different cell states (Gheller et al., 2020). The m^6^A-specific RNA-sequencing revealed a distinct profile of m^6^A modification between proliferating and differentiating C2C12 myoblasts (Gheller et al., 2020). Overexpression of *Mettl3* positively regulated m^6^A RNA methylation and further promoted myogenic differentiation in myoblasts (Chen et al., 2019), while knockdown of *Mettl3* could reduce the mRNA levels of myogenic transcription factor MyoD (Kudou et al., 2017), indicating METTL3 is a positive regulator of skeletal muscle differentiation, which was also confirmed in mice higher or lower expression of METTL3 (Liang et al., 2021). However, a recent study demonstrated that METTL3 might possess negatively regulatory effect on skeletal muscle differentiation. The muscle specific miRNAs, important regulators of skeletal muscle development and muscle cell functions, were significantly repressed by *Mettl3* in C2C12 myoblasts and *in vivo* model of mouse skeletal muscle regeneration after injury, exhibiting an anti-differentiation role of METTL3 in myogenic differentiation (Diao et al., 2021). The conflicting results observed above revealed that the effects of m^6^A modification on proliferation and differentiation in C2C12 myoblasts may also correlated with the cell state, which is in conformed to the observations in ESCs (Geula et al., 2015). Mechanical exploration revealed that the Notch signaling pathway is markedly involved in regulating the muscle stem cells and muscle regeneration of METTL3-mediated m^6^A modification (Liang et al., 2021). METTL14 is another key m^6^A methyltransferase verified to be involved in the prenatal myogenic differentiation (Zhang et al., 2020), which form a stable heterodimeric core complex with METTL3 to regulate m^6^A RNA methylation (Liu et al., 2014). *Mettl14* knockdown could inhibit the differentiation and enhance the proliferation of C2C12 myoblast cells (Zhang et al., 2020).

#### Erasers

Increasing evidences demonstrated that the demethylase FTO significantly promoted myogenic differentiation. In *Fto*-silenced C2C12 myoblasts and *Fto*-deficient mice, the myoblasts differentiation and skeletal muscle development were impaired, respectively, which was associated with mTOR-PGC-1α pathway-mediated mitochondria biogenesis (Wang et al., 2017). In goat primary myoblasts, silencing *Fto* drastically reduced cyclin D1 expression *via* YTHDF2-mediated mRNA degradation, thus resulting in delayed G1 phase and impaired myoblast proliferation (Deng et al., 2021). Moreover, in myoblasts from female embryo, FTO promoted proliferation and myoblast differentiation through the focal adhesion pathway (Huang et al., 2020).

#### Readers

The m^6^A reader protein IGF2BP1 also participated in the regulation of prenatal myogenesis, which target the key marker genes *MYH2* and *MyoG* to promote the differentiation and inhibit the proliferation of C2C12 myoblast cells (Zhang et al., 2020).

### The Role of m^6^A in Neural Stem Cells

Neurodegenerative diseases, including Alzheimer’s disease, Parkinson’s disease, and Huntington’s disease, are highly disabling and greatly influence both patients and their caregivers. However, no curative treatment is available to stop or reverse the disease progression. Increasing evidences have confirmed that the neural stem cells (NSCs), a life-long source of neurons and glia, possess a remarkable promise for the treatment of neurodegenerative diseases because of its beneficial effects such as decrease of neuroinflammation, production of neurotrophic factors, augment of neuronal plasticity and cell replacement (Gage Fred and Temple, 2013; De Gioia et al., 2020). Differentiation of NSCs is labeled by immature differentiational marker *Dcx*, and mature differentiational markers *NeuN* and *Tuj1* (Li et al., 2017; Wang Y. et al., 2018). M^6^A modification seems to be highest in the brain and significantly impacts the cell biological processes in nervous system (Meyer and Jaffrey, 2014; Li J. et al., 2019). Recent studies have implied that the disordered m^6^A modification may be greatly correlated with neurodegenerative diseases (Yen and Chen, 2021). Therefore, regulating the level of m^6^A in NSCs is of great potential for the treatment of neurodegenerative diseases (Table 1).

#### Writers

The m^6^A methyltransferase METTL3 and METTL14 formed a heterodimer to regulate neurogenesis and neuronal development. In 2018, Wang et al. found that the mouse NSCs (mNSCs) defected in *Mettl14* displayed marked decrease in proliferation and premature differentiation, demonstrating that m^6^A modification augmented NSC self-renewal, which was correlated with the alteration of histone modifications including H3K27me3, H3K27ac and H3K4me3 (Wang Y. et al., 2018). In adult mNSCs, *Mettl3* depletion suppressed neuronal development, and skewed the NSCs to differentiate more toward glial lineage by modulating histone methyltransferase Ezh2 and the consequent H3K27me3 levels (Chen J et al., 2019). In zebrafish spinal cord injury model, the m^6^A RNA methylation profiling and transcription level of METTL3 were both increased, which is consistent with the results observed in NSCs in mice with spinal cord injury, indicating METTL3 may contribute to the spinal cord regeneration (Xing et al., 2021).

#### Erasers

The demethylase FTO is abundant in the brain (Mctaggart et al., 2011), its knockout caused m^6^A over-accumulation and damaged neuronal activity (Hess et al., 2013). In *Fto* knockout mice, diminished adult NSCs pool and reduced proliferation and differentiation of adult NSCs was observed, leading to impaired learning and memory. Further exploration of the underlying mechanisms revealed that FTO-mediated m^6^A modification was associated with brain derived neurotrophic factor pathway (Li et al., 2017). However, a recent study revealed that the *Fto* ablation in adult NSCs transiently presented increased proliferation of NSCs and enhanced neuronal differentiation both *in vitro* and *in vivo*. But in a long time, the adult neurogenesis and neuronal development were inhibited by the *Fto* deficiency. Mechanical investigation showed that *Fto* ablation significantly promoted m^6^A modification in Pdgfra and Socs5, which synergistically modulated the phosphorylation of Stat3, therefore repairing the neurogenic deficits induced by *Fto* depletion (Cao et al., 2020).

#### Readers

FMRP is verified to affect the NSCs differentiation and neural development. both the *Mettl14c* and *Fmr1* deficiency in mice caused the nuclear retention of m^6^A-modified FMRP target mRNAs, thus displaying delayed cycle progression of NSCs and extended maintenance of proliferating NSCs into postnatal stages, which indicates FMRP plays a critical role in mediating m^6^A-dependent mRNA nuclear export during neural differentiation (Edens et al., 2019).

### The Role of m^6^A in Bone Marrow Mesenchymal Stem Cells

Bone marrow mesenchymal stem cells (BMSCs), the progenitors for osteoblasts and marrow adipocytes, are commonly obtained from bone marrow aspirates and have been widely considered as the most promising cellular source for tissue regeneration (Baker et al., 2015). Differentiation of BMSCs primarily include osteogenic differentiation and adipogenic differentiation, with *Runx2*, *Sp7*, *Alp*, and *Bglap*, and *Ppary*, *Cebpa*, *Adipoq*, *Plin1*, and *CD36* as markers, respectively (Wu et al., 2018). With widespread ethical acceptability and relative accessibility, BMSCs are the most commonly used mesenchymal stem cells (MSCs) in clinical trials (Zhou et al., 2019).

#### Writers

The existed researches about the m^6^A RNA methylation in BMSCs mainly focused on their osteogenic and adipogenic differentiation capacity. METTL3-mediated m^6^A modification make significant contributions to the bone formation, osteogenic differentiation, and prevention of increased marrow adiposity *via* multiple mechanisms (Wu et al., 2018; Tian et al., 2019; Yan et al., 2020; Peng et al., 2022). *Mettl3* knockout significantly decreased the translation efficiency of MSCs lineage allocator parathyroid hormone receptor-1 (Pth1r) and suppressed the parathyroid hormone (PTH)-caused osteogenic and adipogenic responses in mice, unveiling METTL3-mediated epigenetic m^6^A RNA methylation in BMSCs from an early phase regulated Pth1r translation and affected their responses to PTH during bone accrual (Wu et al., 2018). During osteogenic induction in BMSCs, it is METTL3 instead of METTL14, FTO and ALIBH5 that was upregulated. Further investigation revealed that *Mettl3* knockdown reduced the level of bone formation-related genes, as well as the mineralized nodules formation, which might be correlated with the involvement of the phosphatidylinositol 3-kinase/AKT (PI3K-Akt) signaling pathway (Tian et al., 2019). In addition, *Mettl3* knockdown also decreased the level of *Vegfa* and its splice variants in BMSCs, indicating METTL3 could promote angiogenesis to improve bone regeneration (Tian et al., 2019). The m^6^A methylation of precursor-miR-320/runt related transcription factor 2 (RUNX2) is another factor controlling the osteogenic differentiation of BMSCs. METTL3 enhanced, whereas silence or knockout of METTL3 decreased, the m^6^A RNA methylations of RUNX2, a key transcription factor for osteoblast differentiation and bone formation, and precursor-miR-320, which was rescued by the downregulation of mature miR-320 (Yan et al., 2020). Furthermore, METTL3 mediated m^6^A methylation of LINC00657, which served as a ceRNA to enhance the expression of BMPR1B *via* sponging miR-144-3p, could promote osteogenic differentiation of BMSCs (Peng et al., 2022). However, as a regulator of m^6^A modification, METTL3 also possessed a negative effect on osteogenic differentiation of BMSCs. METTL3 markedly enhanced m^6^A RNA methylation to MYD88 and subsequently activating NF-κB which is widely considered as a suppressor of osteogenesis (Yu et al., 2020). In the process of adipogenic differentiation, METTL3 expression negatively regulated adipogenic differentiation of porcine BMSCs *via* the JAK1/STAT5/C/EBPβ pathway in an m^6^A-YTHDF2-dependent manner (Yao et al., 2019). Moreover, METTL3 inhibited expression of AKT protein in BMSCs by mediating m^6^A RNA methylation of AKT1, thus reducing MSC adipogenesis and alleviating chemoresistance in AML cells (Pan et al., 2021). METTL14 was also involved in the proliferation and osteogenic differentiation of BMSCs, which triggered the m^6^A RNA methylation of PTPN6 to increase its mRNA stability and activated Wnt signaling pathway to enhance cell proliferation and osteogenic differentiation (Cheng et al., 2021).

#### Erasers

The m^6^A demethylase ALKBH5 presented a crucial role in inhibiting MSCs osteogenic capacity by improving the mRNA decay rate of protein arginine methyltransferase 6 (PRMT6) and discovered the PI3K/AKT pathway as a pivotal downstream target of the ALKBH5-PRMT6 axis (Li Z. et al., 2021). ALKBH5 could also dynamically reverse the METTL3-induced m^6^A methylation, and coordinated with METTL3 to regulate osteogenic differentiation *via* NF-κB signaling (Yu et al., 2020). Additionally, ALKBH5 promoted osteoblast differentiation through modulating *Runx2* mRNA stability (Feng et al., 2021). The m^6^A demethylase FTO also possesses a crucial role in BMSCs differentiation, the expression of which was improved during adipocyte differentiation whereas its expression was reduced during osteoblast differentiation, indicating FTO facilitated BMSCs to differentiate into adipocytes compared with osteoblasts (Shen et al., 2018). Mechanical investigation demonstrated that FTO bound to the mRNA of Peroxisome proliferator-activated receptor gamma (PPARγ) and increased its expression, the knockdown of which further blocked the function of growth differentiation factor 11 (GDF11)-FTO to inhibit osteoblast differentiation in BMSCs. Therefore, downregulation of the GDF11-FTO-PPARγ axis might be beneficial for increasing bone formation instead of adipogenesis to treat osteoporosis (Shen et al., 2018). Furthermore, FTO could also trigger osteoporosis of BMSCs through demethylating *Runx2* mRNA and suppressing osteogenic differentiation (Wang J. et al., 2021).

#### Readers

YTHDF1 and YTHDF2 are important readers involved in the proliferation and differentiation of BMSCs. YTHDF1 targeted the zinc finger protein ZNF839, which interacted with and augmented the transcription activity of *Runx2*, and further enhances osteogenic differentiation of BMSCs (Liu T. et al., 2021). YTHDF2 played a crucial role in METTL3-mediated adipogenic differentiation, which facilitated the degradation of JAK1 to inhibit JAK/STAT signaling pathway, thus suppressing the differentiation of BMSCs (Yao et al., 2019).

### The Role of m^6^A in Hematopoietic Stem Cells

Hematopoietic stem cells (HSCs) are a population of cells possessing significant self-renewal ability and multipotent differentiation to all blood cell types (Li Z. et al., 2019). HSCs therefore act as an important cell type both in the clinic and in basic studies, where HSC transplantation is extensively used for a number of malignant and non-malignant diseases, including leukemia (Wilkinson and Nakauchi, 2020). Myeloid differentiation of HSCs is associated with various markers, including *CD11b*, *CD14*, *Gr1*, and *Mac1* (Vu et al., 2017; Lee et al., 2019). Emerging evidences have emphasized the significance of m^6^A RNA modification in maintaining stem cell function in normal and abnormal hematopoiesis, indicating regulation of m^6^A RNA methylation in HSCs possessed pivotal role in determining its fate and further affecting its usage in treating diseases such as acute myeloid leukaemia (AML) (Weng et al., 2019; Wang P. et al., 2021).

#### Writers

In AML cells, expression of METTL3 mRNA and protein are more abundant than in healthy HSCs or other types of tumor cells, the depletion of which promotes cell differentiation and apoptosis and delays leukemia progression in mice (Vu et al., 2017). The direct target of m^6^A in HSCs was *Myc*, a marker of HSC symmetric and asymmetric commitment. The translation of *c-Myc* mRNAs could be improved in the human AML MOLM-13 cell line, while *Mettl3*-deficient HSCs failed to upregulate MYC expression following stimulation of differentiation (Vu et al., 2017; Lee et al., 2019). In addition, loss of *Mettl3* also resulted in enhanced levels of phosphorylated AKT and further promotes the differential effects of *Mettl3* depletion (Vu et al., 2017). Yao et, al. reported that METTL3 dominated the function of METTL3-METTL14 m^6^A methyltransferase complex in catalyzing m^6^A formation by promoting the expression of genes that maintain HSC quiescence. Nevertheless, METTL14 expression was also regarded to take pivotal role in normal myelopoiesis and AML pathogenesis, the knockdown of which significantly promoted HSCs differentiation into myeloid cells through SPI1-METTL14-MYB/MYC signaling axis (Weng et al., 2018).

#### Readers

YTHDF2 is a critical m^6^A reader associated with the self-renewal and regeneration of HSCs. Various studies demonstrated that suppression of YTHDF2 could bolster HSCs expansion without apparent signs of hematological malignancies, making it a promising way for HSC-based gene therapy (Wang H. et al., 2018; Huang and Broxmeyer, 2018; Li et al., 2018). Mechanical exploration revealed that YTHDF2 deficiency simultaneously inhibited the degradation of mRNAs of Wnt target genes as well as survival and proliferation-related genes, thus augmenting the regenerative capacity of HSCs (Wang H. et al., 2018; Huang and Broxmeyer, 2018; Li et al., 2018). Furthermore, in line with myeloid bias, YTHDF2 expression is induced by inflammatory stimulation in HSCs, and deletion of which leads to proinflammatory pathways activation (Mapperley et al., 2021).

## Problems and Pespectives

The derivation of iPSCs has almost revolutionized stem-cell research, which not only possess the self-renewal and differentiate capacity like ESCs, but also display a set of advantages because of generating from somatic cells, including freedom from ethical debates, establishments of patient-derived models of disease for etiology research and disease treatment, and important resources for experimental transplantation therapies (Rowe and Daley, 2019). In comparison to ESCs and iPSCs, adult stem cells exhibit restricted potency, but display advantages such as less concern regarding the tumorigenicity and similar gene-expression pattern to adult cells (Blau and Daley, 2019).

Increasing evidences have proved that stem cells therapy is efficient in regenerating damaged tissues of diseases, including heart failure, diabetes, and cerebral palsy (Gurusamy et al., 2018; Blau and Daley, 2019). As reviewed above, m^6^A modification plays a critical role in facilitating SCT by regulating the proliferation and differentiation capacity of stem cells to affect embryonic development, osteogenesis, adipogenesis, myogenesis, hematopoiesis, neurogenesis and neuronal development (Cao et al., 2020; He and He, 2021). m^6^A marks a wide range of transcripts associated with self-renewal and differentiation of stem cells, the dynamic changes of which need not only maintain cells self-renewal capacity, but also require the flexibility to differentiate upon receiving corresponding stimulus (Batista et al., 2014; Geula et al., 2015). M^6^A modification always leads to different outcomes in the same or different types of stem cells owning to the differences in species or tissue sources, cell states, culture environment, and so on (Kudou et al., 2017; Chen et al., 2019; Gheller et al., 2020; Diao et al., 2021; Liang et al., 2021). Therefore, more researches are required to clarify the underlying mechanisms in different kinds of stem cells. M^6^A RNA modification regulates proliferation and differentiation of stem cells by various mechanisms, mainly affecting RNA metabolism such as RNA stability, translation, decay, splicing, transport, and processing. In overall, m^6^A methylation promotes the translation and decay, reduces the mRNA stability of targeted genes. Interestingly, some differences are observed in BMSCs that m^6^A modification increases the mRNA stability of targets in the process of adipogenic differentiation (Yao et al., 2019), while decreasing the mRNA stability of corresponding genes to trigger osteogenic differentiation (Yan et al., 2020; Cheng et al., 2021). However, more researches are required to verify the correctness and elucidate the behind mechanisms. Additionally, a series of fundamental questions should be in careful consideration in order to fully clarify the effect of m^6^A in various biological processes of stem cells: 1) New components associated with m^6^A modification, such as writers, erasers, readers, and regulatory proteins need to be identified to further clarify the networks of m^6^A. 2) The lineage-specific genes are closely associated with stem cells differentiation, which might be the targets of m^6^A modification to enable direct differentiation of stem cells into required lineages. For example, both the writer METTL3, and the erasers FTO and ALKBH5 can target the *Runx2* mRNA of BMSCs to regulate osteogenesis, indicating the methylated level of *Runx2* mRNA is pivotal for osteogenic differentiation (Yan et al., 2020; Wang J. et al., 2021; Feng et al., 2021). However, more efforts are needed to determine the key genes associated with the cells differentiation and clarify the potential mechanisms. 3) Although the m^6^A demethylase FTO and ALKBH5 are reported to facilitate leukemogenesis, their effects on proliferation and differentiation of HSCs have not been investigated. However, FTO and ALKBH5 demethylate m^6^A RNA to promote the mRNA stability and affect nuclei-to-cytoplasm transport of targets to regulate the proliferation and differentiation of stem cells such as ESCs and BMSCs (Chen X. et al., 2021; Li Z. et al., 2021; Han et al., 2021). More researches are in requirement to elucidate the role of FTO and ALKBH5 in the cell fate determination of HSCs, thus facilitating their application in treating diseases such as AML. 4) As the adipose-derived stem cells (ADSCs) and BMSCs are of increasing importance in the application of SCT to treat diseases of the cardiovascular, nervous and sports systems (Venkataramana et al., 2010; Ahmed et al., 2014; Xu et al., 2016) owning to their characteristics such as multi-directional differentiation, low immunogenicity and high portability (Miao et al., 2017), researches assessing the effects of m^6^A in ADSCs and BMSCs are more required; 5) The transition of islet cells, myocardial cells and chondrocytes from stem cells is of great significance for the treatment of diabetes, heart diseases and osteoarthritis, more studies are needed to determine the role of m^6^A in the above processes; 6) researches investigating the regulation of m^6^A in stem cells have been limited mainly to proliferation and differentiation, despite the fact that other functions of stem cells, such as migratory, homing, immunomodulatory, anti-inflammatory and anti-fibrotic functions, are of the utmost importance for SCT; 7) m^6^A modification is regulated by a variety of functional proteins. To date, limited research has been conducted on ALKBH5, YTHDF1, YTHDC1 and the HNRNPC family, which may modulate stem cell proliferation and differentiation.

## Conclusion

In conclusion, dynamic changes in m^6^A sites or levels regulated by the coordination between methyltransferase complexes and demethylase complexes alter the m^6^A landscape in stem cells.

Elucidating the detailed mechanisms and functional roles of m^6^A RNA in the differentiation and self-renewal processes of stem cells will be essential for treating diseases. As reading proteins bind specific m^6^A-RNA, m^6^A indirectly or directly enhances or impedes the expression of key genes responsible for stem cell proliferation and differentiation (Ji et al., 2018). However, the study of m^6^A in SCT is still in its infancy, and much research remains to be conducted to facilitate its application.
